# Cloning and function analysis of a *Saussurea involucrata LEA4* gene

**DOI:** 10.3389/fpls.2022.957133

**Published:** 2022-07-19

**Authors:** Hui Kong, Wenwen Xia, Mengjuan Hou, Nan Ruan, Jin Li, Jianbo Zhu

**Affiliations:** ^1^Key Laboratory of Agricultural Biotechnology, College of Life Sciences, Shihezi University, Shihezi, China; ^2^Biotechnology Research Institute, Chinese Academy of Agricultural Sciences, Beijing, China; ^3^Hainan Yazhou Bay Seed Laboratory, Sanya, China

**Keywords:** *Saussurea involucrate*, *SiLEA4*, low temperature stress, transgenic, tomato, *E. coli*, photosynthesis, yield

## Abstract

Late embryogenesis abundant proteins (LEA) help adapt to adverse low-temperature environments. The *Saussurea involucrate SiLEA4*, which encodes a membrane protein, was significantly up-regulated in response to low temperature stress. *Escherichia coli* expressing SiLEA4 showed enhanced low-temperature tolerance, as evident from the significantly higher survival numbers and growth rates at low temperatures. Moreover, tomato strains expressing SiLEA4 had significantly greater freezing resistance, due to a significant increase in the antioxidase activities and proline content. Furthermore, they had higher yields due to higher water utilization and photosynthetic efficiency under the same water and fertilizer conditions. Thus, expressing SiLEA4 has multiple advantages: (1) mitigating chilling injury, (2) increasing yields, and (3) water-saving, which also indicates the great potential of the *SiLEA4* for breeding applications.

## Introduction

Coldstress, which includes chilling (<20°C) and freezing (<0°C) injuries, affecting both plant growth and geographical distribution, is one of main factors restricting crop yield and quality (Chinnusamy et al., 2007). Many economically important crops such as cotton, corn, peppers, rice, soybeans, tomatoes, some tropical fruits (e.g., bananas, papayas, and mangoes), and subtropical fruits (e.g., grapes, oranges) are sensitive to low temperature stress. The unpredictable inversion events that accompany late spring directly contribute to low seedling survival in these crops. Early frost events in early fall and winter lead to early crop mortality, causing crop yield reductions while greatly reducing the quality of produce (Sharma et al., 2005). Also, in cold climate areas, frost damage is a chronic problem for crop production. Therefore, it is highly significant to study the cold tolerance genes for the genetic improvement of crops.

The main regulatory pathway of plant cold stress response is the ICE (Inducer of CBF expression)-CBFs (C-repeat/DREB binding factors)-COR (Cold-regulated) network, where COR is the key determinant of this response. The key COR genes that determine plant cold stress response contain the late embryogenesis abundant proteins (LEA) family (Shi et al., 2018). LEA proteins are a large stress-inducible protein family that exists widely in plants and are involved in plant abiotic stress responses. They were first identified during cotton embryo development and abundantly accumulated during late seed maturation (Dure et al., 1981). LEA proteins have since been reported in seeds and in nutrient tissues of various species under different stress conditions (Ingram and Bartels, 1996; Thomashow, 1999). Expression of some LEA genes can enhance stress resistance in bacteria, yeast and plants (Xu et al., 1996; Houde et al., 2004; Puhakainen et al., 2004; Zhao et al., 2011; Jaspard et al., 2012; Liu et al., 2014,Liu et al., 2015,Liu et al., 2016; Wang et al., 2014,Wang et al., 2017; Du et al., 2016; Sharma et al., 2016; Zhou et al., 2017). Members of the LEA family subgroup IV all share a characteristic ∼75 amino acid long N-terminal sequence and a conserved region that is helps against plant water stress (Hu et al., 2012). For example, tomato Group IV LEA proteins accumulate abundantly in leaves and help reduce intracellular water loss (Zegzouti et al., 1997). Under drought stress, the group IV in *Arabidopsis* can maintain lactate dehydrogenase activity through altered conception (Raynal et al., 1999). Silencing of group IV LEA genes in peanuts reduces its drought tolerance (Su et al., 2011).

Tomato (*Solanum lycopersicum* L.) is a typical chilling-sensitive vegetable crop (Ré et al., 2017), whereas *Arabidopsis* has greater cold tolerance. Both express similar CBF proteins against cold stress, but share only four similar proteins downstream of the CBF response, thereby suggesting that the downstream genes are more important for tomato cold sensitivity. Most studies on freezing stress tolerance have focused on upstream regulation of cold response (Chinnusamy et al., 2003; Hu et al., 2013; Ding et al., 2015; Kim et al., 2015; Ma et al., 2015; Li H. et al., 2017), but few studies have been conducted on downstream genes. Contrastingly, *S. involucrata* have evolved a complex set of cold tolerance mechanisms comprising important cold tolerance genes, due to their unique growth environment. Therefore, using the cold-sensitive tomato as a model plant to study the cold tolerance genes of *S. involucrata* is quite novel. In previous studies, expression of the *SiPIP5A* gene improved the low-temperature tolerance in tomato by regulating the cell water balance (Li et al., 2020). The *SiDHN* gene expression can promote cold and drought tolerance of transgenic tomato plants by inhibiting cell membrane damage, protecting the chloroplasts, and enhancing the reactive oxygen species (ROS) scavenging capacity (Ganapathi et al., 2019).

Recently, transcriptome sequencing and extensive bioinformatics analysis were completed for *S. involucrata*, thus providing us with an opportunity to study its cold stress tolerance-specific LEA genes (Li J. et al., 2017). A significantly up-regulated *SiLEA4* gene expression was identified based on the *S. involucrata* low temperature stress transcriptomic data (not publicly available). In the present study, we cloned the *SiLEA4* from the *S. involucrata* cDNA template. This study expressed *SiLEA4* in both *E. coli* and tomato plants and then studied the low temperature tolerance of *E. coli*. By evaluating agronomic traits, changes of photosynthetic parameters, morphological and physio-biochemical characteristics of the tomato plants, this study implies that the *SiLEA4* may be important in conferring microorganisms and plants low temperature stress resistance.

## Materials and Methods

### Plant materials and growth conditions

Tomato (*L. esculentum*) variety “Yaxin 87-5” was used for genetic transformation in this study, and its seeds were provided by Yaxin Seed Co. Ltd, Shihezi City, Xinjiang, China. Tomato seeds were sterilized and grown on a 1/2 MS medium in a group culture room at 26°C with approximately 60% humidity and a 14/10 h light/dark cycle. The transformed tomato seedlings, on reaching ∼10 cm, they were hardened and then placed in a natural light climate chamber at 30°C and about 60% humidity. T_2_ transgenic tomato lines were screened with 50 mg/L kanamycin (Kan), and putative transgenic plants were selected by basic phenotypic and PCR evaluation. The confirmed positive plants were subjected to low temperature treatment at 4 and −2°C. The *Saussurea* tissue culture seedlings are provided by our laboratory.

### SiLEA4 gene cloning

Total RNA was extracted from low temperature-treated *Saussurea involucrata* leaves using the RNAisoPlus kit (TianGen Biotech, Beijing, China), and cDNA was synthesized by the cDNA synthesis kit (TRAN, Beijing, China). Using the *SiLEA4* sequence information, gene-specific primers *SiLEA4*-F and *SiLEA4*-R were designed using the Primer Premier 5.0 software (Table 1). PCR products were identified *via* agarose gel electrophoresis, and subsequently purified and cloned into the pMD19T vector (TaKaRa, Dalian, China). It was then transformed into *E. coli* DH5α competent cells, and the positive clones were screened by PCR and verified by DNA sequencing.

**TABLE 1:** The primer used in this study.

*SiLEA4*-F	CCCGGG AGCCACCGACAAACCCTATG	Gene cloning
*SiLEA4*-R	GTCGAC TGCCAGAATGATTCGCCAGT	
*SilEA4*-eGFP F	GCTCTAGATGGCATCTCAACAAGATAAG	Subcellular localization
*SilEA4*-eGFP R	ACTTCTGGAGGGCATAGGTATATGTATATAATGGTGAGCAAGGGCG	
PET28a-F	GGATATCCATATGGCATCTCAACAAGATAAG	Prokaryotic expression
PET28a -R	GGAATTCTATATACATATACCTATGCCCTCCAGAAGT	
RT-*SiLEA4*-F	CGGAGGCACTGAAGAAATCG	qRT-PCR
RT-*SiLEA4*-R	GCCAGAATGATTCGCCAGTT	
Actin28-F	GGTAACATTGTGCTCAGTGGTGG	
Actin28-R	AACGACCTTAATCTTCATGCTGC	

### Bioinformatics analysis

We downloaded 51 LEA protein amino acid sequences from the *Arabidopsis* database and *SiLEA4* gene homologs were identified using Blast P in NCBI. The LEA protein sequences of 12 species, including *Artemisia annua* (PWA93397.1), *Citrus sinensis* (XP_015386949.1), *Daucus carota* (BAJ08390.1), *Olea europaea* var. *sylvestris* (XP_022863156.1), *Pistacia vera* (XP_031252570.1), *Quercus lobata* (XP_030927649.1), *Betula platyphylla* (AST13894.1), *Eucalyptus grandis* (XP_039174169.1), *Eutrema salsugineum* (XP_006410837.1), *Brassica napus* (XP_013679752.1), *Brassica rapa* (XP_009148762.1), and *Lupinus albus* (KAE9621787.1), were found. The amino acid sequences of these species were aligned with the SilEA4 amino acid sequences using ClustalW, and then a rootless evolutionary tree was constructed using MEGA6.0 to determine the nomenclature of the *SilEA4* gene and the homology relationship with other species. The evolutionary tree was embellished using the online tool “Interactive Tree Of Life.” The Conserved Domains Database tool on the NCBI^1^ was used to predict the conserved domains of the SiLEA4 protein. The overall hydrophilicity indicated by the Grand average of hydropathicity index (GRAVY) value of the SiLEA4 protein was predicted by the Expasy program.^2^

#### Prokaryotic expression

Using the above cloned *SiLEA4* gene as a template, PCR amplification was performed using gene-specific primers containing the *EcoR* I and *Nde* I (TaKaRa, Dalian, China) double restriction sites. The *SiLEA4* gene open reading frame (ORF) was subcloned into the pET28a vector, and the recombinant plasmid pET28a-*SiLEA4* was constructed. The coding region and linkage sequence of pET28a were verified by DNA sequencing. The pET28a-SiLEA4 and empty pET28a (−) vectors were separately transformed into the *E. coli* BL21 expression strain using a hot water bath and the transformants were screened using 50 mg/L Kan after incubation at 37°C for 13 h. Overnight *E. coli* cultures from selected colonies were diluted 1,000-fold using fresh LB medium and incubate at 37°C with shaking until OD_600_ = 0.4–1 (preferably OD_600_ = 0.6). Isopropyl b-D-thiogalactopyranoside (IPTG) was then added at a final concentration of 1 mM, and incubated continuously at 37°C for 0, 2, 4, and 6 h. The induced bacterial cells were pelleted down by centrifuging at 6,000 rpm for 10 min, then re-suspended with 40 μl of double distilled water, and finally 40 μl of sample buffer (sucrose, bromophenol blue, 10% SDS solution, 5% β -mercaptoethanol and 0.05 mol/L Tris–HCl (pH = 8.0)) was added to the mix, and boiled in a 100°C water bath for 10 min to collect the clarified cell extract for SDS–PAGE analysis. For the chilling treatment, *E. coli* cultures containing empty pET28a or pET28a-*SiLEA4*, were adjusted to OD_600_ = 0.2 and incubated separately with 0.2 mM IPTG at 4°C with 180 rpm shaking, and equal samples were taken every 2 h to monitor the OD_600_ values, up to 12 h. Under freezing stress treatment, 0.2 mM IPTG was also added to the above cell cultures and then placed in a refrigerator at −2°C for 24 h. After diluting the cell cultures by 10×, 100×, and 1000×, respectively, 2 μl of each sample was taken and plated onto LB plates and incubated overnight at 37°C to observe the growth status of the colonies.

### Subcellular localization

To observe the subcellular localization of the SiLEA4 protein, *Xba* I and *Pst* I digestion sites were added to the ORF of *SiLEA4* cDNA *via* gene-specific primers containing these restriction sites. PCR amplification was performed using a high fidelity DNA polymerase. The PCR product was cloned into the N-terminal end of the *eGFP* gene under the control of the cauliflower mosaic virus 35S (CaMV 35S) promoter to obtain the pCAMBIA2300-*SiLEA4*-e*GFP* subcellular localization fusion expression vector. The pCAMBIA2300-e*GFP* and the pCAMBIA2300-*SiLEA4*-e*GFP* vectors were separately transformed into *Agrobacterium tumefaciens* strain GV3101 through electroporation using the GENE PULSER II system. The pCAMBIA2300-e*GFP* was injected into the lower epidermis of fully expanded leaves of tobacco and incubated for 48 h in the dark, for observing the subcellular localization by confocal scanning microscopy (NIKON, C2_+_, Japan).

### Plant expression vector constructs and plant transformation

To demonstrate the function of *SiLEA4* in plant low temperature resistance, we constructed a plant expression vector. To generate a pCAMBIA2300-*SiLEA4* recombinant plasmid, the *SiLEA4* ORF was cloned into the pCAMBIA2300 vector under the control of the *CaMV35S* promoter. The insert was released from pMD19-T-*SiLEA4* through *Sma* I and *Sal* I digestion, and then ligated into pCAMBIA2300 MCS. Constructs were introduced into *Agrobacterium* strain GV3101 *via* electroporation.

This study deliberately chose the cold-sensitive plant tomato to evaluate the effects produced by the *SiLEA4*. *SiLEA4* was transferred into tomato by *Agrobacterium*-mediated leaf disc transformation. Infected tomato hypocotyls were screened on the MS medium containing 2.0 mg/L 6-BA, 0.3 mg/L IAA, 100 mg/L Kan, and 400 mg/L timentin (Tm). The selected resistant transgenic shoots were transferred to the rooting medium (1/2 MS medium containing 0.3 mg/L IAA, 100 mg/L Kan, and 400 mg/L Tm). Transgenic tomato seedlings were identified and verified *via* PCR by using DNA from transgenic plants as a template, pCAMBIA2300-*SiLEA4* recombinant plasmid as the positive control, and wild type (WT) tomato plant DNA as the negative control. The *SiLEA4* gene expression was confirmed by RT-PCR. Transgenic plants were transplanted into plastic flower pots containing nutrient soil and vermiculite (3:1) and grown in a naturally lit greenhouse at 22–28°C with 60–70% relative humidity until flowering and fruiting. The T_2_ generation seeds of transgenic tomato were collected for further analysis.

### Measurement of physiological and biochemical indexes under chilling and freezing stresses

To assess the effect of cold stress on *SiLEA4* expressing tomato plants, WT and T_2_ transgenic tomato plants grown at 25°C for 30 days, were transferred to a 4°C incubator for 6 h, followed by a further 4 h in a −2°C incubator. Changes in tomato phenotypes were observed at different periods and tomato leaves were collected separately for physiological index measurements. The relative water content (RWC) of the leaves was determined by the weighing method. Two blades weighing 1 g were accurately weighed, and the fresh weight (FW) of the sample was recorded as 1 g. One leaf was soaked in distilled water for 70 min, dried with absorbent paper, and the saturated fresh weight (TW) of the leaf was determined. The other leaf was placed in a kraft paper bag, placed in an oven at 80°C, and rec-orded as dry weight (DW) after being weighed several times and kept unchanged. RWC was calculated as RWC = (FW-DW)/(TW-FW), and each treatment was repeat-ed three times. The relative electrolyte leakage (REL) was measured as per the method of (Jungklang et al., 2017), using an EC 215 conductivity meter (Markson Science Inc., Del Mar, CA, United States). REL was calculated using the following equation: REL (%) = L1/L2 × 100%, where L1 is the conductivity value of the soaking solution, and L2 is the conductivity value after boiling. Malondialdehyde (MDA) content was measured according to the thiobarbituric acid (TBA) method. Fresh leaves were weighed to 0.1–0.15 g and ground in liquid nitrogen, 400 μL of 5% TCA was added and centrifuged at 6,000 r/min for 15 min at 4°C. 400 μL of supernatant was transferred to a new centrifuge tube, 0.5% TBA dissolved in 5% trichloroacetic acid TCA was added to 1 mL, mixed thoroughly and boiled in a boiling water bath for 30 min. The mixture was cooled to room temperature on ice and centrifuged at 5,000 r/min for 15 min. Absorbance values were measured at 600 nm, 532 nm and 450 nm and the MDA content was calculated as C(mol/L) = 6.45 (*A_*532*_–A_*600*_*) –0.56 (*A_*450*_*) (Sekmen et al., 2012). The absorbance was determined using UV1901PC ultraviolet spectrophotometer (Shanghai AoXi Company), the same below. Free proline content was determined by the sulfosalicylic acid method, 0.2–0.3 g of fresh tomato plant leaves were added to 3 mL of 3% sulfosalicylic acid solution, boiled for 10 min, and filtered. The filtrate was added with 2 mL of glacial acetic acid and 2 mL of 2.5% acidic ninhydrin solution, and boiling water was boiled for 30 min. The solution was cooled and 4 mL of toluene was added, shaken and mixed thoroughly, allowed to stratify and then the upper layer was pipetted into a new 10 mL centrifuge tube at 3,000 r⋅min^–1^ for 5 min. The absorbance values were compared to a standard curve constructed using a known amount of proline. Soluble sugars were determined by the anthrone method (Bates et al., 1973). 0.1–0.15 g of fresh leaves were added to 10 mL of distilled water, boiled for 20 min, cooled to room temperature and then fixed to 100 mL. One milliliter of the abrove solution was added to 5 mL of 0.2% anthrone solution and the absorbance values were measured at 625 nm. The absorbance values were compared with the constructed glucose standard curve. The soluble protein content was analyzed according to the (Gu et al., 2013). 0.1–0.15 g of tomato leaves were weighed and ground into a homogenate with 5 mL of distilled water or buffer solution, centrifuged at 3,000 r/min for 10 min and the supernatant was set aside. Take 1.0 mL of the sample extract and add 5 mL of Coomassie Brilliant Blue reagent, shake it well, and the absorbance was measured at 593 nm after 2 min. The protein content was checked by glucose standard curve. Superoxide dismutase (SOD), peroxidase (POD), catalase (CAT), and ascorbate peroxidase (APX) activities were determined as previously described (Nakano and Asada, 1980; Cakmak et al., 1993; Polle et al., 1994). Firstly, the enzyme solution was prepared. The 0.1 g sample (fresh leaves as appropriate) was washed and placed in a pre-cooled mortar. The 1.6 ml 50 mmol/L pH7.8 pre-cooled phosphoric acid buffer (PBS) was added and ground into a homogenate on an ice bath, transferred into a centrifuge tube at 4°C and centrifuged at 12,000 rpm for 20 min, the supernatant was the enzyme solution. The activity of SOD enzyme was determined by nitrogen blue tetrazole (NBT) reduction method. Briefly, 3 mL of reaction mixture (162 mL Met solution, 0.6 mL EDTA-Na2 solution, 5.4 mL PBS, 6 mL NBT solution, 6 mL riboflavin solution) and 30 μL enzyme solution were taken, respectively. The tubes were placed in a light incubator and reacted for 20 min under 4,000 Lux light. Two tubes were made at the same time. In one tube, 3 ml of reaction mixture was added to 30 μl PBS (without enzyme solution), which was measured as the maximum photoreduction tube after being illuminated, and the other tube was added with buffer solution only and placed in the dark place for zero setting. The absorbance value at 560 nm was measured by avoiding light after zero setting with the unilluminated contraption tube. The activity of POD was determined by guaiacol method. 3 ml of reaction solution (200 mL 0.2 M pH 6.0 PBS, 0.076 mL guaiacol, 0.112 mL 30% H_2_O_2_) was taken, and 30 μL of enzyme solution was added, zero was set with PBS as control, and then the absorbance value at 470 nm was measured (determination for 60 s). CAT activity was determined. 3 mL of the reaction solution (200 mL 0.15 M, pH7.0 PBS, 0.3092 mL 30% H_2_O_2_) was added into 0.1 mL of the enzyme solution. The absorption value under 240 nm was measured (60S determination) by zero setting with PBS as the control. For the activity of APX, 0.10 mL of enzyme solution was taken, and 1.70 mL of PBS containing 0.1 M MEDTA-Na_2_ (0.05 mol/L, pH7.0) was added, then 0.10 mL of 5 mm ASA was added, and finally 0.10 mL of 20 mM H_2_O_2_ was added. Immediate determination of OD_290_ at 20°C within 40 s. All samples were tested in triplicates.

### Determination of agronomic traits and photosynthetic capacity

The agronomic characteristics of the transgenic tomato plants were evaluated in Shihezi, Xinjiang, China (N44° 20′, E85° 30′). Both transgenic and WT tomatoes were planted in an area of 10.5 m^2^ and three biological replicates were used for each experiment. First, plant height, branch diameter, and root length were measured for both transgenic and WT plants. The distance between the base of the main stem and the tip of the stem was calculated as the plant height (cm). The distance between the base of the main stem and the tip of the main root was calculated as the root length (cm). Branch diameter (sd) was measured on the soil surface using a digital vernier caliper. Subsequently, the FW of the plants was measured and then placed in kraft paper bags, placed in an oven at 105°C for 30 min, then dried to a constant weight at 80°C and the DW was measured. For the fruit yield assessment, we investigated the number of individual fruit plants and the weight of both the individual fruit and individual fruit plants. Finally, this study measured the cross and longitudinal diameter of the tomato fruit.

Fully expanded leaves of four tomato independent transformation events and three WT tomatoes were measured for photosynthetic parameters by the GFS-3000 photosynthetic apparatus at 9:45–10:55 h on a sunny day. Three instantaneous photosynthetic values were recorded for each leaf, for the photosynthetic parameters, including transpiration rate (Tr), net photosynthetic rate (Pn), intercellular CO_2_ concentration (Ci), stomatal conductance (Gs), PSII maximum fluorescence efficiency (Fv/Fm), and water use efficiency (WUE). WUE is calculated as follows: WUE = Pn/Tr.

### Statistical analysis

All data were initially organized using Excel 2010. SPSS 18.0 and GraphPad Prism 8.0 software were used for statistical analysis. Duncan’s multiple comparison tests was used to determine significant differences between the wild-type and transgenic lines. A significance level of *p < 0.05 indicates a significant difference. ^**^p < 0.1 indicates extremely significant differences. Error bars in all figures represent the standard deviations from the mean.

## Results

### Sequence analysis of SiLEA4 from *Saussurea involucrata*

**FIGURE 1:**
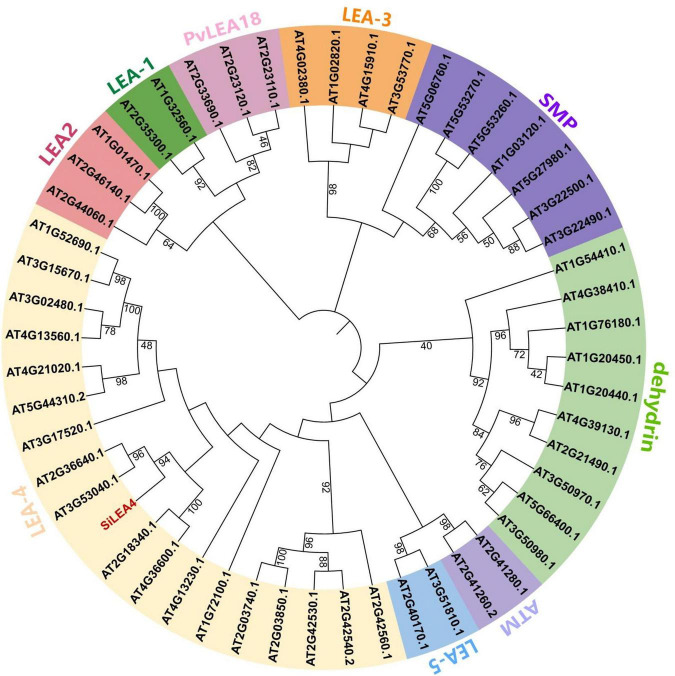
Phylogenetic analysis of *SiLEA4* gene. LEA proteins of other plant species included *Arabidopsis thaliana*, *Artemisia annua* (PWA93397.1), *Citrus sinensis* (XP_015386949.1), *Daucus carota* (BAJ08390.1), *Olea europaea* var. *Sylvestris* (XP_022863156.1), *Pistacia vera* (XP_031252570.1), *Quercus lobata* (XP_030927649.1), *Betula platyphylla* (AST13894.1), *Eucalyptus grandis* (XP_039174169.1), *Eutrema salsugineum* (XP_006410837.1), *Brassica napus* (XP_013679752.1), *Brassica rapa* (XP_009148762.1), *Lupinus albus* (KAE9621787.1) with *Saussurea involucrata LEA4* unrooted phylogenetic tree. The phylogenetic tree was built using MEGA 6.0 by the Neighbor-Joining method with 1000 bootstrap replicates.

Based on our previous research, we observed the *SiLEA4* expression being rapidly up-regulated at low temperatures. To further investigate the potential function of this gene at low temperatures, we cloned this gene. The *SiLEA4* ORF length is 1,034 bp, and it encodes a protein of 344 amino acids. We conducted a phylogenetic analysis for the *SiLEA4* nomenclature and to understand the function of its homologs. Both NCBI conserved domain analysis of the SiLEA4 protein and phylogenetic tree analysis of its sequence with the *Arabidopsis* LEA protein family indicated that SiLEA4 belongs to the LEA-4 family; hence, the gene was named as *SiLEA4*. Hydrophilic analysis of the SiLEA4 protein revealed that it had significantly more hydrophilic amino acids (negative values) than hydrophobic amino acids (positive values), which indicated it as a hydrophilic protein. Phylogenetic tree analysis of the SiLEA4 protein sequence with homologous sequences from other species revealed that the protein is most closely related to its homolog from *Artemisia annua* (Figure 1). However, no clear functional annotation was found for this homolog.

### Expression of SiLEA4 protein improves low temperature tolerance in *Escherichia coli*

**FIGURE 2:**
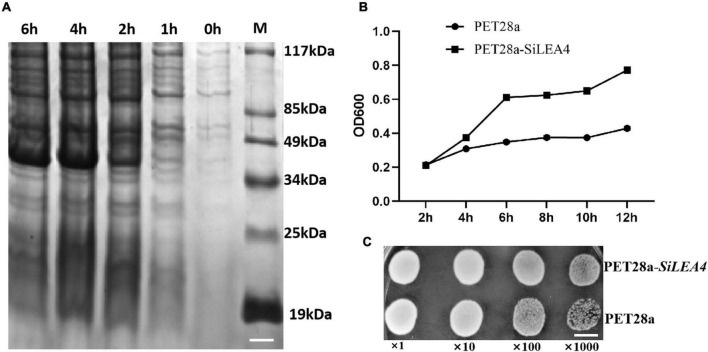
Functional analysis of *SiLEA4* overexpression in *Escherichia coli* BL21 for low temperature tolerance. (A) Induced expression of the *SiLEA4* (PET28a-*SiLEA4*) protein in *E. coli*. 0, 1, 2, 4, 6 h: the IPTG induction times, respectively. *M*. low molecular-weight protein marker. Bar = 1 cm. (B) Growth kinetics of *E. coli* transformed with PET28a (control) and PET28a-*SiLEA4*. The bacteria were cultured at 4°C and 180 rpm. The OD600 values were measured every 2 h to evaluate the growth conditions. (C) The growth performance of *E. coli* DE3 (PET28a, upper)/(PET28a-SiLEA4, down) after stress –2°C. The cell cultures were adjusted to OD600 = 1. Cells were growth on –2°C for 24 h and were then diluted serially (1:10, 1:100, 1:1000). Two microliters of each sample was spotted onto the LB plates and then incubated for 8 h for 37°C. Bar = 80 mm.

This study hypothesized that the SiLEA4 expression would induce low temperature resistance in *E. coli* due to the conserved nature of this class of proteins in plants and microorganisms. Therefore, we constructed a prokaryotic expression system for SiLEA4. We studied the physicochemical properties of the SiLEA4 protein by transforming pET28a-*SiLEA4* into the *E. coli* BL21, and induced with IPTG. The profile of SiLEA4 protein showed that the expressed protein was ∼41 kDa, which is ∼4 kDa higher than the predicted value of 37.26 kDa (Figure 2A). The SiLEA4 protein accumulation maximized after 6 h of IPTG induction. To assess whether SiLEA4 protein confers the ability to tolerate low temperatures *in vivo*, we exposed the SiLEA4-expressing *E. coli* cells to both cold (4°C) and freezing (−2°C) stresses. Under 4°C growth conditions, the growth rate of the SiLEA4-expressing *E. coli* cells increased rapidly with time, as compared to the *E. coli* cells expressing the empty control vector (pET28a) (Figure 2B). Meanwhile, under −2°C stress, the SiLEA4-expressing cells produced more colonies than the control cells (pET28a) when grown on solid medium at 100 and 1,000-fold dilutions (Figure 2C).

### SiLEA4 is localized to the cytoplasmic membrane

**FIGURE 3:**
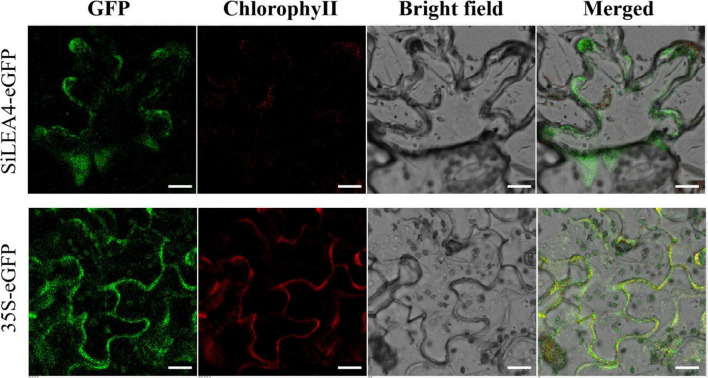
Subcellular location of SiLEA4. SiLEA4 was fused to *eGFP* and transiently expressed in 3-week-old tobacco leaf cells. The empty vector 35S-*eGFP* was used as a control. A green fluorescent signal was observed with confocal microscopy 48 h after *Agrobacterium* infiltration. Bar = 18 μm.

WoLF PSORT^3^ and the Plant-PLoc program^4^ predicted that the SiLEA4 protein may be localized in different cellular locations, including the cell membrane, cytoplasm, and endoplasmic reticulum. To better identify the cellular localization of SiLEA4 protein expression, we constructed a fusion expression vector pCAMBIA2300-*SiLEA4*-*eGFP*, while we used the pCAMBIA2300-e*GFP* as a control. Using laser confocal scanning microscopy, this study found that the pCAMBIA2300-e*GFP* emitted green fluorescence throughout the cells, while it appeared on the cell membrane for the pCAMBIA2300-*SiLEA4*-e*GFP*, thus indicating that SiLEA4 was indeed cell membrane-localized (Figure 3).

### Morphological and physiological changes in SiLEA4-overexpressing transgenic tomato plants under low temperature stress

**FIGURE 4:**
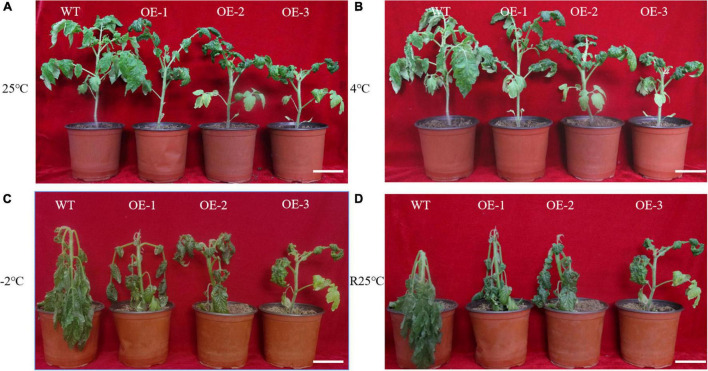
Phenotypes of wild-type and *SiLEA4*-overexpressing transgenic tomato plant under cold stress. (A) Wild-type and transgenic tomatoes were grown in a greenhouse at 25°C for 5 weeks of age. (B) Growth status of tomato plants incubated in a low temperature incubator at 4°C for 6 h. (C) Morphological differences between transgenic and wild-type tomato plants incubated at –2°C for 4 h. (D) Morphological differences between transgenic plants and wild-type plants 1 day after recovery from low temperature stress at 25°C. Bar = 7 cm.

To show that *SiLEA4* conferred low temperature tolerance in tomato, SiLEA4-expressing transgenic tomato lines were cultured at 4, −2, and 25°C. The transgenic plants had shorter height and the leaves were greener in color than the WT plants (Figure 4A). After 6 h exposure to 4°C, the leaves of the transgenic plants showed no obvious appearance of cold damage and the wild-type plants showed only slight leaf drooping (Figure 4B). After exposure to −2°C for 4 h, the WT tomato leaves wilted severely, while only the OE-1 transgenic plants showed leaf wilting (Figure 4C). When re-incubated at 25°C, a proportion of the transgenic plants regained their upright state, whereas the WT plants did not recover and showed severe plant lodging (Figure 4D).

**FIGURE 5:**
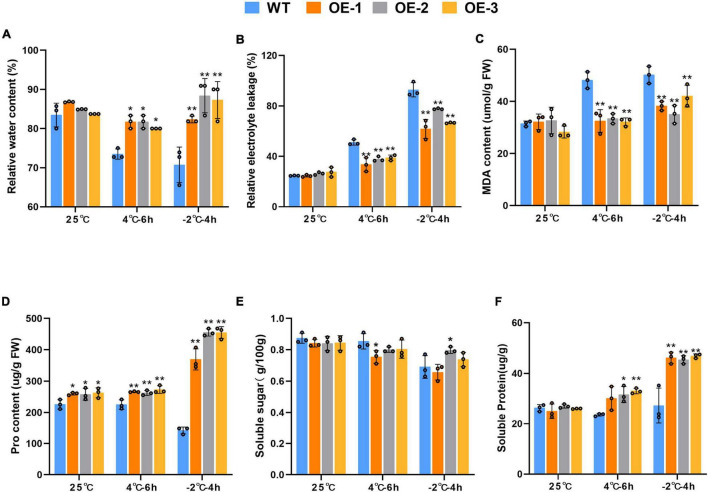
Physiological changes of wild-type in wild-type and SiLEA4-overexpressing transgenic tomato plant lines (OE-1, OE-2, and OE-3) under cold stress. (A) RWC (%), (B) REL (%), (C) MDA content, (D) pro content, (E) soluble sugar, (F) soluble protein. Data are means ± SD of three replicates. Asterisk(s) indicate significant difference between the wild-type and transgenic plants: *P < 0.05 and **P < 0.01.

Before stress exposure, RWC levels in the leaves of the transgenic and WT plants were similar. After exposure to the 4°C stress, although the RWC levels decreased in both WT and transgenic plants, the transgenic RWC levels were significantly higher than in the WT plants. However, the RWC of transgenic plants of the OE-2 and OE-3 strains were extremely significantly higher than that in WT plants under −2°C stress (Figure 5A). MDA, a ROS-induced lipid peroxidation byproduct, together with REL, reflects the extent of plasma membrane damage to the. Under cold stress, both WT and transgenic plants showed an increase in MDA content and REL levels under cold stress, but the increase was more pronounced in the WT plants. At 4 or −2°C, both REL and MDA were extremely significantly higher in the WT plants than in the transgenic plants (*p* < 0.01) (Figures 5B,C). The proline content is partly indicates the plant’s resistance under adversity. Proline and soluble protein contents were also highly significantly higher under low temperature stress. It is noteworthy that the transgenic plants had higher proline content than the WT plants at 25°C. At 4 and −2°C, the transgenic plants both proline and soluble protein contents of being higher than those of the WT plants (*p* < 0.01) (Figures 5D,F). However, there was no significant difference in soluble sugar content between WT plants and transgenic plants at room temperature or low temperature (Figure 5E).

**FIGURE 6:**
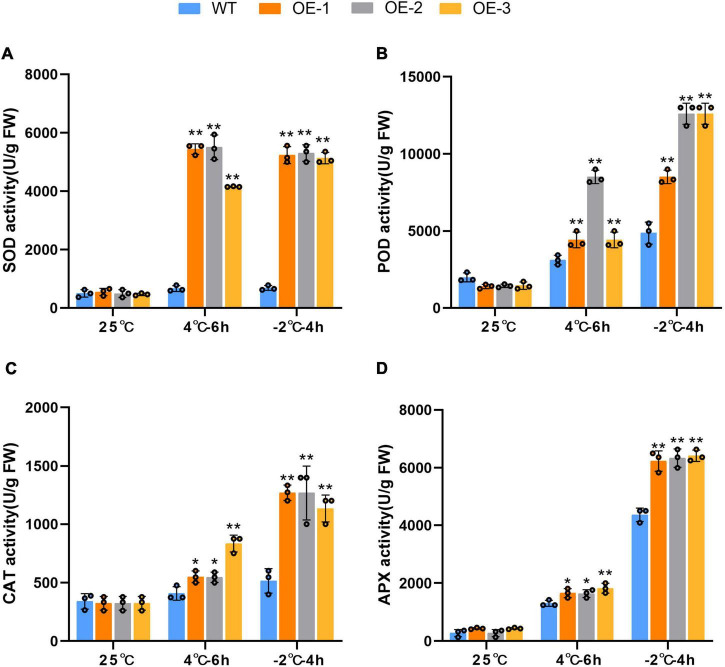
Comparison of related enzymatic activities in leaves of transgenic and wild-type plants under normal and cold stress conditions. Comparison of SOD activity (A), POD activity (B), CAT activity (C) and APX activity (D) in leaves of transgenic and wild-type plants under normal and cold stress conditions. WT represents the wild-type tomato plants. OE-1, OE-2, and OE-3 represent three independent *SiLEA4* transgenic tomato lines. Data are means ± SD of three replicates. Asterisk(s) indicate significant difference between the wild-type and transgenic plants: *P < 0.05 and **P < 0.01.

To evaluate the ROS scavenging enzyme activities of the transgenic *SiLEA4* plants, we studied the enzymatic activities of APX, CAT, SOD, and POX (Figure 6). The results showed that the activities of all four enzymes were up-regulated in both WT and transgenic plants under low temperature stress. The dramatic increase in the SOD activity in transgenic tomatoes under 4°C stress was the most significant by being 7.54 times higher than in the WT. In addition, the activity of POD was extremely significantly higher than that of WT plants, while the activity of CAT and APX was also higher than that of WT plants. The activities of all four enzymes, SOD, POD, CAT, and APX, were significantly up-regulated under −2°C stress, with mean values of 5.29, 2.31, 2.37, and 1.55 times higher than those of WT plants, respectively. This indicates that the transgenic plants have a strong ROS scavenging ability.

### Changes in agronomic traits and photosynthetic capacity of transgenic tomato plants

**FIGURE 7:**
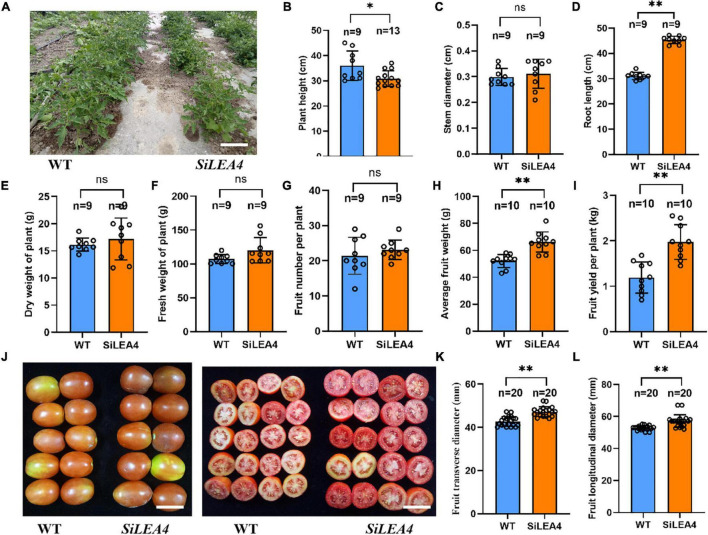
Agronomic traits analysis of *SiLEA4* transgenic tomato. (A) Performance of 8-week-old *SiLEA4* transgenic and wild-type tomato plants in field. Bar = 25 cm. (B–F) Comparison of plant height, Stem diameter, root length, plant fresh and dry weight between *SiLEA4*-transformed plants and wild-type plants. (G–I) Tomato yield of transgenic line and wild type. (J) The whole fruit appearance (left) and cross section (right) of transgenic lines and wild type. Bar = 50 mm. (K,L) Comparison of transgenic tomato fruit with wild-type tomato fruit in cross-sectional and longitudinal diameter. Error bars, mean ± SD. *n* = Number of plants. ns. no significant difference. The asterisks indicate a statistically significant difference (two-tailed Student’s *t*-test, *P < 0.05, ^**^P < 0.01).

**FIGURE 8:**
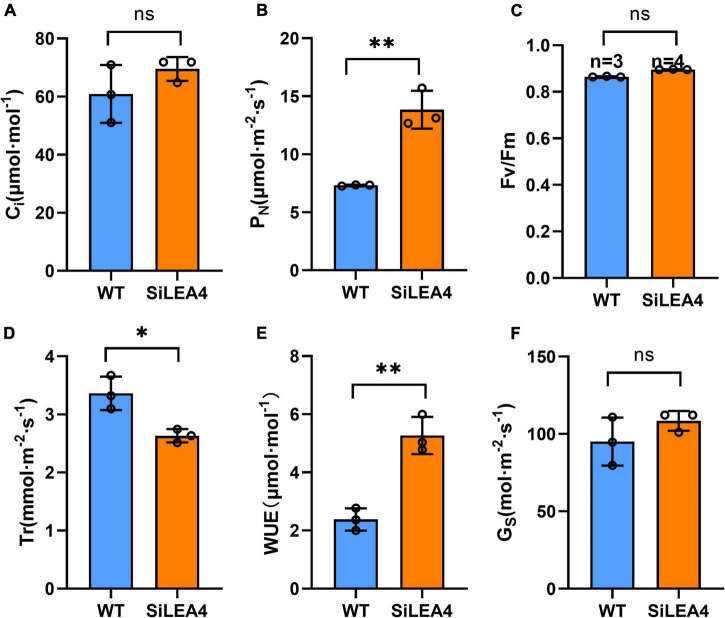
Photosynthetic capacity analysis of wild-type tomato and transgenic tomato. (A) Intercellular CO_2_ concentration; (B) net photosynthetic rate; (C) Fv/Fm values; (D) transpiration rate; (E) water use efficiency; (F) stomatal conductance. Error bars, mean ± SD. *n* = Number of plants. ns. no significant difference. The asterisks indicate a statistically significant difference (two-tailed Student’s *t*-test, ^**^P < 0.01).

The results of the agronomic traits of the transgenic and WT tomatoes are shown in Figure 7. At 90 days of normal growth, the transgenic plants were shorter and had longer roots than the WT tomatoes. There were no significant differences in FW, DW, or the number of fruits between the GM and WT tomato plants. However, the weight per fruit of the transgenic tomatoes was higher than that of the WT tomatoes. In fruit yield measurements, the average yield of the transgenic plants was also significantly higher than in the WT plants. Additionally, in the photosynthetic capacity measurements of the transgenic and WT tomatoes, the results showed that the Pn of the WT tomato plants was 7.322 μmol⋅m^–2⋅^s^–1^, E was 3.364 mmol m^–2⋅^s^–1^, Ci was 60.92 μmol⋅m^–1^, Gs was 95.042 mol⋅m^–2⋅^s^–1^, and WUE was 2.381 μmol⋅m^–1^. The Fv/Fm values and Ci values were not significantly different from each other (Figure 8).

## Discussion

In this study, we cloned and analyzed the SiLEA4 in *S. involucrata*, which was rapidly up-regulated against cold stress. We established both prokaryotic and plant expression systems for the SiLEA4 protein. This paper explored whether the *SiLEA4* would impact the growth and development of other microorganisms and plants in response to abiotic stresses.

This study found that the cold stress tolerance ability of the *SiLEA4* expressing *E. coli* was enhanced, thereby implying that the SiLEA4 protein may confer chilling and freezing resistance to *E. coli* cells. In the previous studies, expression of the *Arabidopsis AtLEA33* conferred cold stress tolerance in *E. coli* (Zhao et al., 2011). Expression of the *Salvia miltiorrhiza SmLEA1* and *SmLEA2* enhanced salt and drought tolerance in *E. coli* (Wang et al., 2017); while the *CsLEA11* expression enhanced its cell viability and improves the heat and cold stress tolerance (Zhou et al., 2017). This is consistent with our findings.

In the present study, transgenic plants expressing SiLEA4 showed low temperature resistance at both 4 and −2°C. This paper used the internal physiological and biochemical changes in tomato to explain the phytomorphological phenotypic changes. This paper argues that the main challenges for plants under freezing stress are water loss and cytoplasmic membrane disruption. RWC reflects the water retention capacity of the plants and it is used to measure the water status and osmotic adjustments of the plants (Larbi and Mekliche, 2004). In this study, the transgenic plants had higher RWC under low temperature conditions compared with wild-type plants, indicating that the *trans-SiLEA4* tomato plants could better maintain water balance and could better sustain metabolic activities to proceed under low temperature conditions. The transgenic tomato had higher proline content and soluble protein content under adversity could well explain its excellent water retention ability (Shafi et al., 2019).

Under 25°C culture conditions, we detected higher proline levels in transgenic tomatoes expressing SiLEA4, suggesting that the SiLEA4 protein may cause elevated proline levels in a relatively direct manner. Lv et al. demonstrated that some LEA proteins can act like transcription factors to increase the expression level of certain drought-resistant genes (Lv et al., 2021). Yu et al. (2016) showed that LEA can act as a molecular chaperone to prevent the descent of two interacting proteins. Therefore, this study speculates that this approach may be the result of SiLEA4 acting as a specific transcription factor and molecular chaperone to increase the expression and activity of proline-related synthase. Transgenic plants expressing SiLEA4 also exhibited higher proline accumulation than wild-type plants under three temperature conditions, 25, 4, and −2°C, with a significant increase in proline accumulation especially at −2°C. The functions of proline water retention, molecular chaperone and ROS reduction have also been confirmed in previous studies (Ronde et al., 2004; Rodriguez and Redman, 2005; Chaves et al., 2008; Verbruggen and Hermans, 2008; Szabados and Savouré, 2010). Bohn et al. (2007) found that the differences in the degree of enhanced frost resistance of different varieties of winter wheat after cold domestication corresponded to the amount of proline accumulation. Abdelaal et al. (2019) found that a certain concentration of proline treatment increased the RWC and the activity of ROS scavenging enzymes under salt stress. These studies are mutually verifiable with the findings of the present study (Abdelaal et al., 2019). Therefore, this paper considers the elevated proline content as the most important evidence to explain the increased cold resistance of *SiLEA4* at the biochemical level.

Soluble protein is one of the important osmoregulatory substance. In this experiment, soluble protein content in tomato plants increased significantly under low-temperature stress, however, the increase in tomatoes of the transgenic SiLEA4 plants under −2°C stress was more significant than that of the wild-type plants by 1.68 times, indicating that the transgenic strain could accumulate soluble protein more rapidly after low-temperature stress to regulate the osmotic pressure balance inside and outside the membrane and enhance the water-holding capacity to maintain the integrity and stability of the cell membrane structure. This phenomenon may occur for three reasons: first, LEA4 is natively accumulated in large amounts as a soluble protein under freezing; second, it acts as a transcription factor or molecular chaperone to enhance the expression of freeze-tolerant-related genes or to enhance the stability of freeze-response-related proteins; and third, it is indirectly achieved through the elevation of proline content.

In this paper, the degree of cell membrane disruption in tomato leaves was assessed by the production of REL and MDA. Compared with the WT, the increase of REL and MDA levels in the transgenic lines was significantly reduced after cold treatment, indicating that the expression of SiLEA4 reduced the degree of cell membrane damage. Again, these results are partly due to the fact that elevated levels of proline and soluble protein maintain osmotic balance inside and outside the cell membrane. However, the oxidative damage to cell membranes by ROS is even more severe. Hara et al. found that the overexpression of CuCOR19 could inhibit the increase in the degree of cell membrane damage in tobacco by enhancing the scavenging ability of hydroxyl radicals and peroxyl radicals (Hara et al., 2004). This is consistent with what we found. Therefore, this paper argues that the more important reason for these results is the enhancement of the ros clearance system.

Under abiotic stress, the stomatal opening of plants is very low, their CO_2_ fixation is blocked, the electron transport chain produces numerous electrons that cannot be transferred to NAPDH^+^ resulting in their transfer to O_2_, thus forming superoxide ions, thus forming ROS which causes peroxidation of cell membrane lipids. The activity of some antioxidant enzymes (e.g., SOD, CAT, POD, and APX) in the ROS scavenging system will increase accordingly to maintain and protect cell membrane integrity and ensure cellular homeostasis (Mittler et al., 2004). In our study, the SOD, POD, APX and CAT activities of the *SiLEA4* expressing plants were significantly higher than in the WT strain under both chilling and freezing conditions, with them being more substantial under freezing conditions. On the one hand, LEA proteins can act as transcription factors to increase the transcription level of ROS scavengers (Yu et al., 2016), while on the other hand, they can act as molecular chaperones and enzyme protectors to maintain the 3D conformation of ROS scavengers at low temperatures (Lv et al., 2021). Therefore, we suggest that the rapid and substantial up-regulation of SOD under cold conditions is important for tomato low temperature resistance. This indicates that the *SiLEA4*-expressing tomato plants have a higher ROS scavenging capacity than the WT plants, resulting in a faster reduction of the associated adverse effects. This also explains the lower MDA content and REL under our test stress conditions. We proposed that the SiLEA4 protein can compensate for the low ROS resistance in tomato due to the lack of downstream freezing stress response proteins of the CBF cold acclimatization signaling pathway (Zhang et al., 2004).

Finally, this study evaluated the agronomic traits and photosynthetic capacity of the transgenic tomato plants. The results showed that the transgenic plants had longer roots, shorter plants, and larger and heavier fruits than the WT plants. Ganapathi et al. showed that expression of the *SiDHN* in the LEA2 group of tomatoes resulted in shorter height, longer root length, heavier roots, higher DW, and thicker stems in the transgenic plants as compared to WT plants (Ganapathi et al., 2019). The shorter plant height and longer root length were presumably due to expression of the SiLEA4 protein, which triggered the stress responses of the tomato (Salleh et al., 2012). These results suggest that some LEA family genes indeed affect the plant growth and development.

In the photosynthetic data, this study found a significant increase in net photosynthetic rate, a significant decrease in net transpiration rate and a highly significant increase in WUE in the expression lines. The shorter height but no change in biomass of the expressed SiLEA4 tomato plants was due to their higher Pn, which resulted in greater dry matter accumulation and transport of these assimilates to the tomato fruit, resulting in an overall larger fruit size overall. Under stress conditions, the photosynthetic system is strengthened and protected by protein modifications like phosphorylation (Aro and Ohad, 2003; Rochaix, 2007; Vener, 2007; Tikkanen et al., 2008,Tikkanen et al., 2010; Liu et al., 2009). We hypothesize that the enhanced photosynthetic rate is due to SiLEA4 acting as a possible stress signaling molecule, which ultimately generates a stress-resistant response. The decrease in transpiration rate is hypothesized to be due to the enhanced water retention capacity of the transgenic tomatoes, and this tends to occur after abiotic stresses. This implies that SiLEA4 has pleiotropic effects in the low temperature resistance. Therefore, the excellent stress-resistance performance of the SiLEA4 warrants further investigation into its mechanisms and causes.

Overall, the cytoplasmic membrane-bound *SiLEA4* improved the low-temperature stress tolerance of *E. coli*. *SiLEA4*-expressing transgenic tomato plants showed higher cold tolerance than WT plants, which was mainly attributed to their significantly higher antioxidant enzyme activity and antioxidants, which greatly improved their ROS scavenging ability and reduced the associated cell membrane damage. Furthermore, the improved Pn and WUE of the transgenic lines allowed the plants to accumulate more organic matter, thus increasing their yield. Thus, our results suggest that the *SiLEA4* has potential applications in cold resistance breeding.

## Data Availability Statement

The original contributions presented in this study are included in the article/Supplementary Material, further inquiries can be directed to the corresponding authors.

## Author Contributions

HK, WX, and MH designed the experiments, analyzed data, and wrote the manuscript text. NR participated in the whole experiment and revised the manuscript. JL and JZ guided the experiments. All authors read and approved the final manuscript.

## Conflict of Interest

The authors declare that the research was conducted in the absence of any commercial or financial relationships that could be construed as a potential conflict of interest.

## Publisher’s Note

All claims expressed in this article are solely those of the authors and do not necessarily represent those of their affiliated organizations, or those of the publisher, the editors and the reviewers. Any product that may be evaluated in this article, or claim that may be made by its manufacturer, is not guaranteed or endorsed by the publisher.

## Footnotes

1
https://www.ncbi.nlm.nih.gov/cdd

2
http://www.expasy.org/tools

3
https://wolfpsort.hgc.jp/

4
http://www.csbio.sjtu.edu.cn/bioinf/plant/
