# Supplementary material for: Cloning and function analysis of a Saussurea involucrata LEA4 gene
Source: Front Plant Sci. 2022 Jul 19;13:957133. doi: 10.3389/fpls.2022.957133 (PMC9343949; doi:10.3389/fpls.2022.957133)
Supplement: Supplementary file 1 [file Data_Sheet_1.docx]

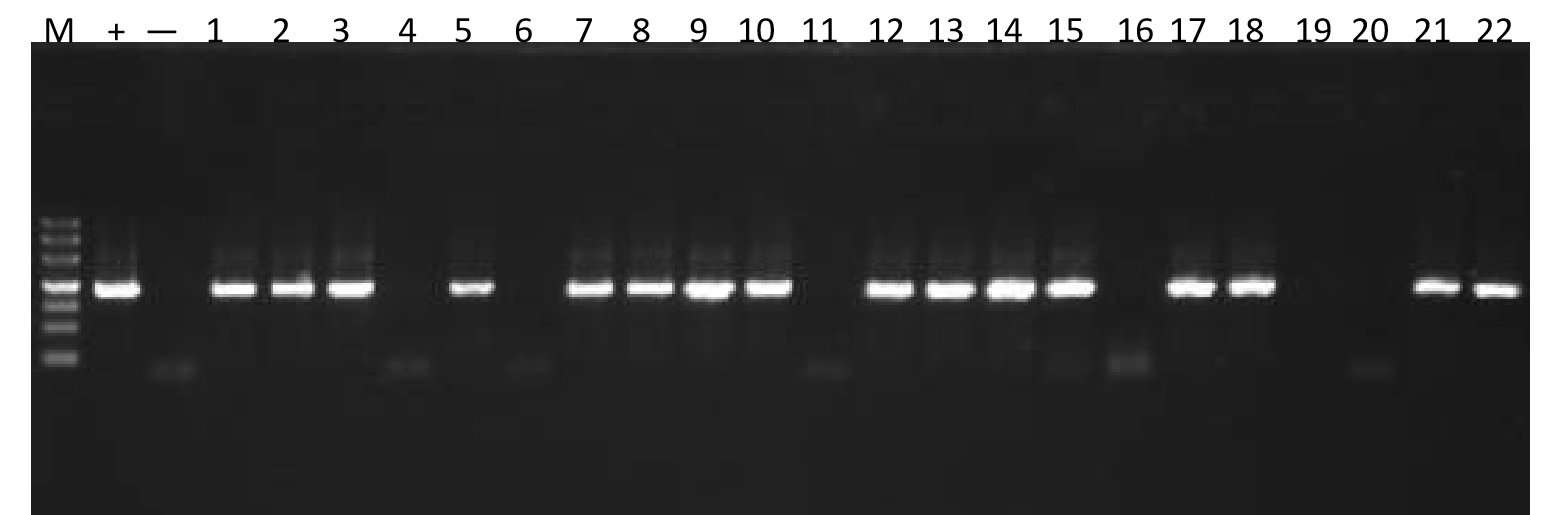


**Supplemental Figure S1.** Characterization of transgenic tomato through DNA PCR. Numbers 1-22 indicate individual transgenic plant lines, ＋ the plasmid used as positive control and - the non-transgenic plants used as negative control.


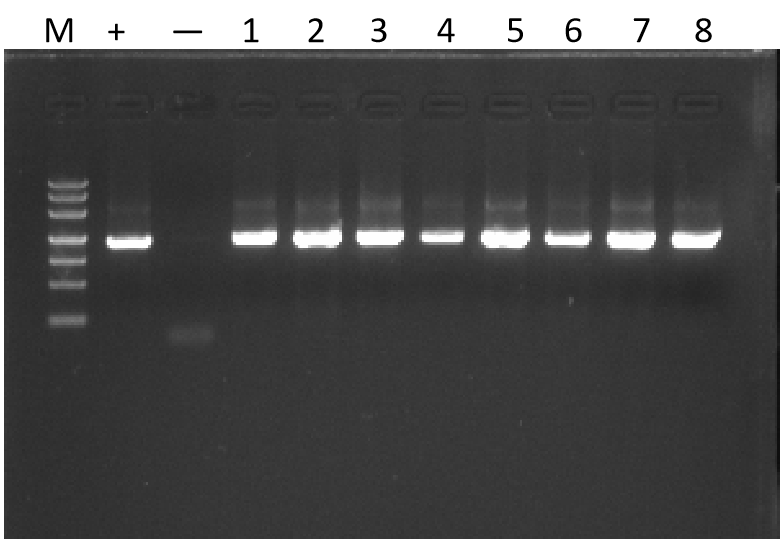


**Supplemental Figure S2.** Characterization of transgenic tomato expressing *SiLEA4* through RT-PCR. Numbers 1-8 indicate individual transgenic plant lines, ＋ the plasmid used as positive control and - the non-transgenic plants used as negative control.


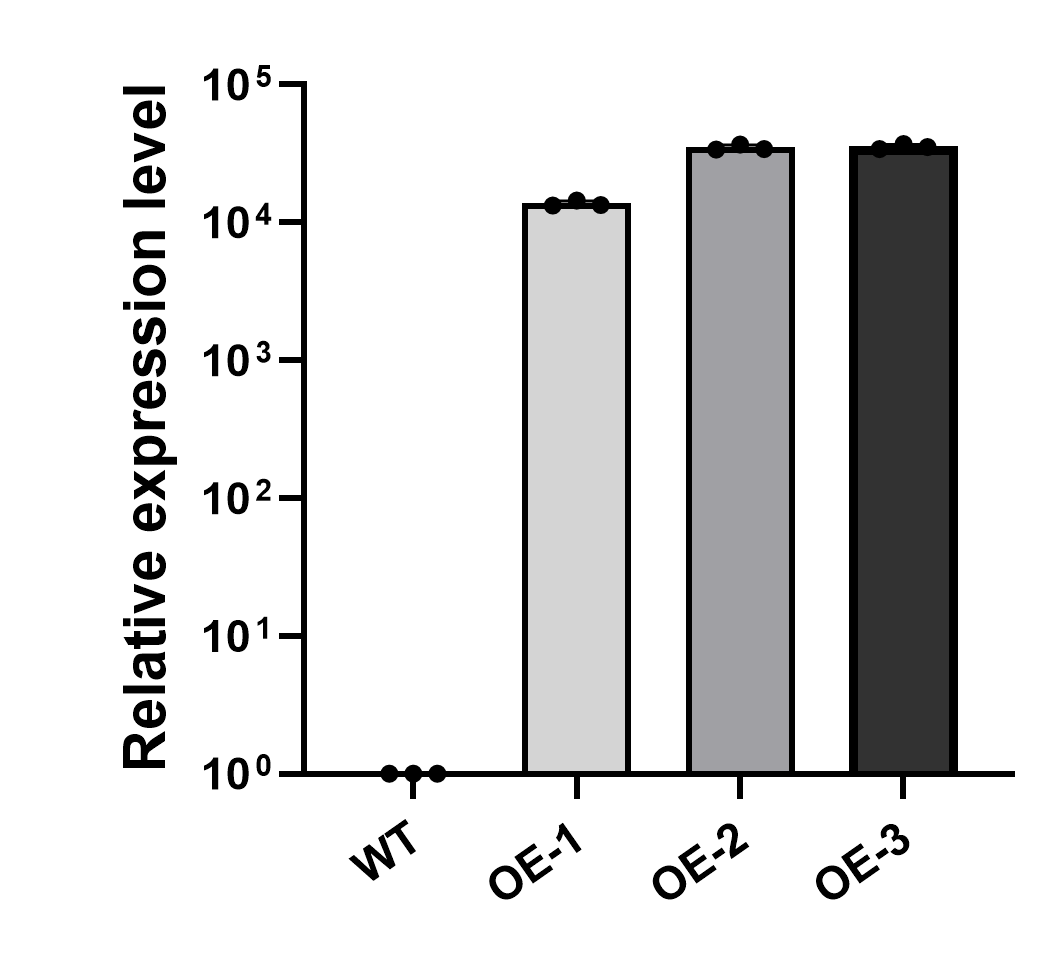


**Supplemental Figure S3**. Characterization of transgenic tomato expressing *SiLEA4* by qRT-PCR. The relative expression data was represented by the 2^-∆∆CT^ and calculated through making the WT as the control.
